# Study on healing technique for weak interlayer and related mechanical properties based on microbially-induced calcium carbonate precipitation

**DOI:** 10.1371/journal.pone.0203834

**Published:** 2018-09-13

**Authors:** Changyu Jin, Dong Liu, Anlin Shao, Xin Zhao, Lei Yang, Fuquan Fan, Kunpeng Yu, Rongbing Lin, Jingzhu Huang, Chenggong Ding

**Affiliations:** 1 Intelligent Mine Research Center, Northeastern University, Shenyang, Liaoning, P. R. China; 2 School of Resource and Civil Engineering, Northeastern University, Shenyang, Liaoning, P. R. China; 3 School of Engineering, RMIT University, Melbourne, Australia; 4 Wuhan Institute of Geodesy of the Chinese Academy of Sciences, Wuhan, Hubei, P. R. China; East China Normal University, CHINA

## Abstract

The weak interlayer refers to the filling material in shear belts or large-scale structural planes, which is usually composed of soil, fine sand and gravels. It is prone to argillization when encountering water and its mechanical strength and stiffness are generally low, which has adverse effects on the stability of underground structures. In recent years, research on reinforcement techniques for weak interlayers has been a hot topic in geotechnical field. As a new reinforcement method for structural planes, the microbial healing technique has attracted a lot of attention. In this paper, a study on the healing technique for weak interlayer based on microbially induced calcium carbonate precipitation (MICP) and related mechanical properties was conducted for the interlayer shear belt at Baihetan Hydropower Station in China. First, *Sporosarcina pasteurii* was activated in laboratory. Reinforcement of the weak interlayer was realized by utilizing calcium carbonate precipitation on the weak interlayer. Continuous monitoring of the precipitates on the weak interlayer by XRD and SEM indicated that the precipitates on the weak layer were microbially induced calcium carbonate. Its crystals were irregular fish scale-shaped cubes with size in the range of 5~20*μ*m. With favorable crystal growth, the crystals and the particles of the weak interlayer were cemented together. Finally, the mechanical properties of the healed weak interlayer were tested and the variations of uniaxial compressive strength, shear strength and triaxial compressive strength with bacteria concentration were discussed. The test results indicated that the maximum uniaxial compressive strength, peak shear strength and triaxial compressive strength can be increased by 149%, 162% and 119%, respectively, which subsequently improve the overall strength of the shear zone or structural plane. This can provide a new idea for soft ground reinforcement in underground projects.

## Introduction

**Fig 1:**
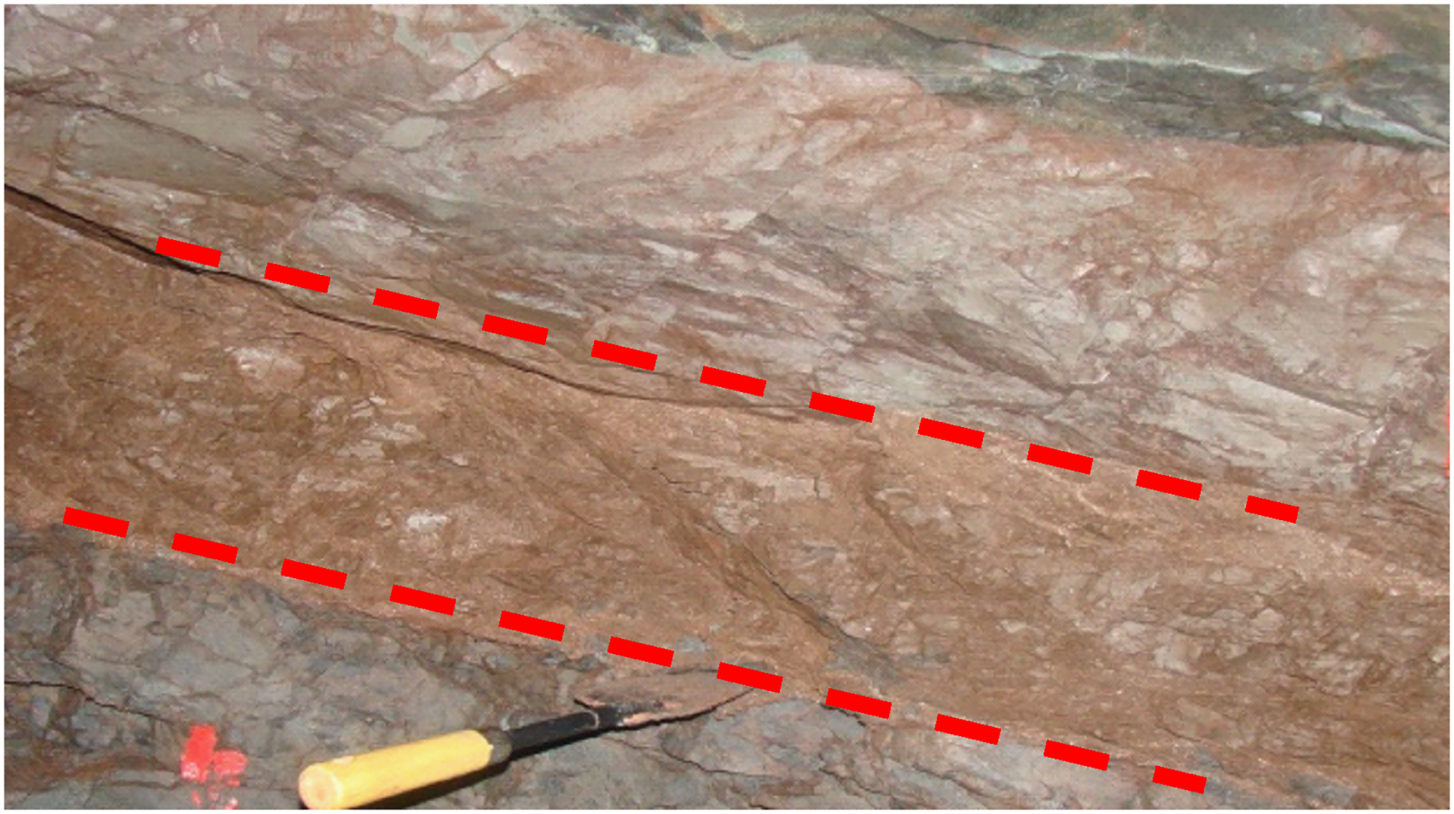
Weak interlayer in rock mass.

During long-term tectonic activities, a great number of weak interlayers are formed in natural rock masses, which greatly hamper rock mass integrity, but also change the mechanical properties of rock masses, as shown in Fig 1. A lot of engineering geological hazards have indicated that the existence of weak interlayers in rock masses usually plays a controlling role in the stability of geotechnical projects. Cracking of underground cavern sidewalls, dislocation of the upper and lower rock strata, roof collapse, landsliding and other hazards may be triggered. In September 2008, a debris flow occurred due to tailings dam break at the Tashan Iron Mine of Xinta Mines Ltd. in Linfen, Shanxi Province, China, resulting in 277 people dead and 4 missing, and a direct economic loss of 96.19 million RMB [1]. In January 2011, flood, landslide and mudflow were triggered by heavy rain in the mountainous region of Rio De Janeiro, Brazil, leading to 806 people dead and 300 missing [2]. In November 2015, a landslide occurred at the jade mine in Sankaco Village in Kachin State of Burma, leading to loss of 113 lives and 100 missing [2]. Therefore, improving the integrity and mechanical strength of weak interlayer is the prerequisite to reduce and avoid the occurrence of engineering geological disasters.

Since 1950s, the experts in geotechnical engineering around the world have started to heal and reinforce rock and soil by chemical means. A classical work by Norwegian scientists Rosenqvist and Bjerrum [3] proved that if a clay is sedimented in salt water and later subjected to a hydraulic gradient resulting in a leaching out of the salt, the undrained shear strength of the clay will be reduced and its sensitivity will increase. If a clay is deposited in fresh water, its shear strength will be two to three times as high as one sedimented in salt water. As a reinforcement technique, many researchers have experimentally studied microbially induced calcium carbonate precipitation (MICP) since 1990s and a number of achievements have been made. Ferris and Setehmeir [4] reduced the permeability of sandy soil by using MICP. It was found that the flow rate in sandy soil decreased by 50% after 45 hours and the sandy soil layer was almost completely sealed after 120 hours. Gollapudi et al. [5] found that, by adding nutrient solution into the mixture of *Bacillus cohnii* and sand, the fissures can be effectively sealed and the permeability was close to 0. Ramakrishnan et al. [6,7], Ramachandran et al. [8] and Jonkers et al. [9] utilized calcium carbonate generated during the MICP process in healing and reinforcement of cracks in concrete. Tiano et al. [10], Stocks-Fisher et al. [11], Castanier et al. [12] and Rodriguez-Navarro et al. [13] indicated that microbially induced calcium carbonate precipitation can effectively protect and heal calcareous stone and investigated the crystalline form of calcium carbonate under different conditions, which was either calcite or aragonite. Whiffin et al [14]. proved that, under the effect of MICP, the soil permeability can be reduced by 22~75%. By repeatedly injecting nutrient solution into cured sand with a 60mL plastic injector for 64 days. Rebata-Landa [15] found that the content of calcium carbonate in the specimen increased with increasing reactant concentration and reaction time. Paassen [16] suggested that the type of calcium carbonate crystals generated during the MICP process was mainly related to the rate of urea hydrolysis and observed the morphology of calcium carbonate crystals formed between sand particles during the MICP process. Meanwhile, the uniaxial compressive strength of cured samples reached 0.7MPa to 12MPa. By the MICP experiments. Chou et al. [17] found that the friction angle of the soil sample cured under high bacteria concentration was larger than that under low bacteria concentration. Achal et al. [18] conducted MICP experiments in cement mortar, and found that a simulated crack of 27.2 mm in the cement mortar was successfully repaired with a porosity reducing over 50% and a compressive strength improving about 40%. The investigation of Bundur et al. [19] showed that *Sporosarcina pasteurii* vegetative cells have a severe retardation effect on hydration kinetics of cement-based materials. Furthermore, after the first day of hydration, the bacterial mortar displayed compressive strength which was similar to or greater than that of the neat mortar. Helle et al. [20] re-established high salt concentration in leached low-saline, highly sensitive clays significantly improves their mechanical properties. The undisturbed shear strength increased from less than 10 to 25–30 kPa, and the remolded shear strength increased from less than 0.5 to more than 6 kPa. Di Maio and Scaringi [21] found that the residual friction angle increase greatly with the pore solution concentration, shear displacements with decreasing rate occurred. On the subsequent exposure to distilled water, the displacement rate increased progressively and the specimens re-experimented failure, the shear strength and average pore ion concentration on the slip surface seemed to be related by the same relation as that evaluated in the absence of chemical gradients. Pontolillo et al. [22] indicated that the specimens didn’t experiment creep when exposed to 1M NaCl solution, on the contrary, exposure to distilled water made the displacement rate increase greatly. The decrease in pore ion concentration obliterated the over-consolidation effects. Mwandira et al. [23] completely removed 1036 mg/L of Pb^2+^ by MICP in the laboratory. These results were further confirmed by scanning electron microscope (SEM) and X-ray diffraction (XRD) analysis, which indicating a coprecipitation phenomenon of calcium carbonate and lead. Li et al. [24] utilized the MICP in MK-modified cement mortars to improve its compressive strength, despite of cement replacement with MK as high as 50%, the technology improved compressive strength of mortars by 27%, which was still comparable to those mortars with 100% cement. The results proved that biomineralization could be effectively used in reducing cement content without compromising compressive strength of mortars. Li et al. [25] investigated the effectiveness of biocement as admixture with fly ash (FA) to improve geotechnical properties of expansive soils. This research indicated that incorporation of biocement in fly ash is an effective means of increasing the strength of expansive soils. The experiments of Liu et al. [26] indicated that the bacterially induced calcium carbonate mineralization can greatly improve the shear stiffness, peak shear strength and residual strength of cracks, and the healing effect depends on time. Zamani et al [27] performed MICP experiments in silty sands containing fines up to 35%, and the results indicated that the effect of MICP on silty sands depends on relative density, fines content, and the fabric governing structure of the soil.

The previous studies mainly focused on the healing and reinforcement technique for rock and soil based on chemical method. Less attention has been paid on MICP healing technique for weak interlayers in rock and soil and the mechanical properties promotion of healed weak interlayers. In this study, the calcium carbonate precipitation induced by *Sporosarcina pasteurii* was applied to heal weak interlayers in rock masses. The calcium carbonate precipitation process was continuously monitored by XRD and SEM. It was found that the CaCO_3_ particles and the weak interlayer particles were cemented together. It was shown that the MICP technique can be applied to heal the weak interlayers in rock masses. Laboratory tests were then conducted to study the mechanical properties of the healed weak interlayer. The role of microbial healing technique in improving the integrity and mechanical performance of weak interlayer was investigated.

## Materials and method

### Composition of weak interlayer

**Fig 2:**
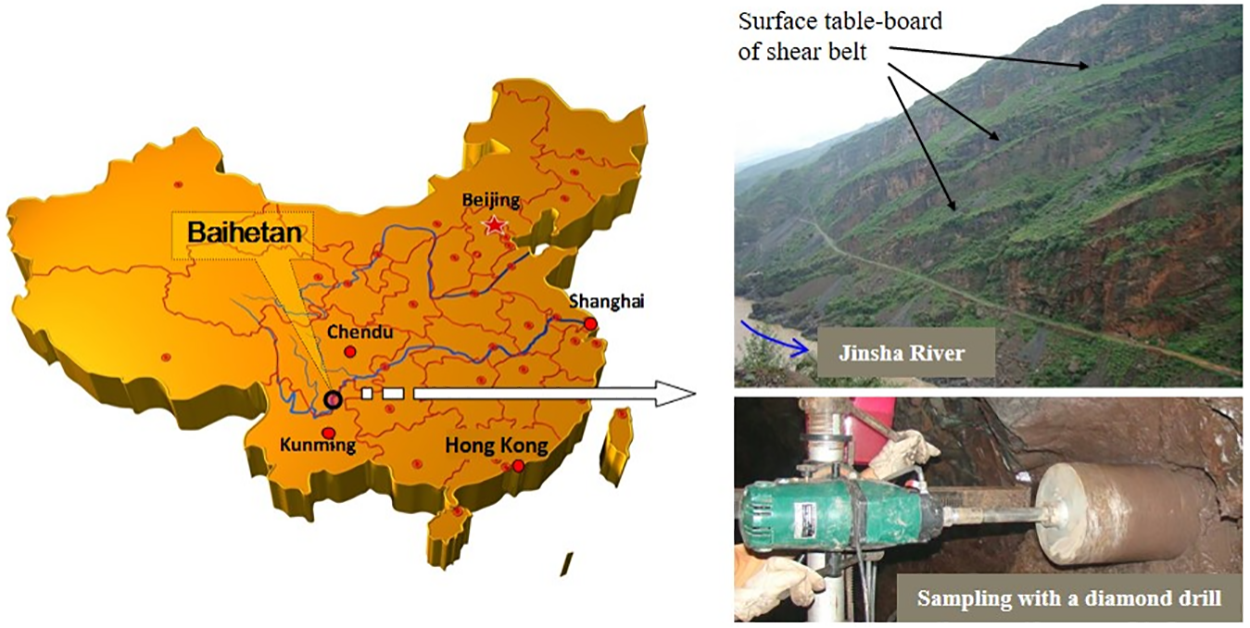
Baihetan Hydropower Station location map and interlayer shear belt sampling.

Our study was approved by Huadong Engineering Corporation Limited (Hang zhou, Zhejiang province, China). The samples used in the laboratory tests were taken from the interlayer shear belt zone C4 at right bank site of Baihetan Hydropower Station located on the lower reach of Jinsha River, China, as shown in Fig 2 (3012489, 588638, 642). The alternative hard and soft rock strata were subjected to shearing during tectonic activities. Under the long-term physical and chemical effects of groundwater, thin interlayer shear belts were formed with poor properties. The XRD analysis was conducted to identify the mineral composition of 5 groups of random samples of the interlayer shear belt zone C4. The analysis results were shown in Table 1. The interlayer shear belt contains clay minerals, accounting for about 25% of the total minerals in the weak interlayer. The clay minerals mainly exist in the form of illite/montmorillonite mixed layer, illite and chlorite. Few of them are in the form of water swellable montmorillonite. The physical parameters of interlayer shear belt were shown in Table 2.

**Table 1:** Mineral composition analysis of interlayer shear belt zone C4.

Number of sample	Mineral composition(%)			Clay minerals composition(%)			
	Hematite	Sphene	Clay minerals	Montmorillonite	Illite mixed layer	Illite	Chlorite
1	37	36	27	0	70	20	10
2	29	43	28	5	65	25	5
3	32	46	22	0	65	30	5
4	35	36	29	15	55	20	10
5	38	40	22	20	50	20	10

**Table 2:** Physical parameters of interlayer shear belt zone C4.

Number of sample	*w*(%)	*ρ*(Mg·m^-3^)		*e*	*S*_r_(%)	*w*_L_(%)	*w*_P_(%)	*I*_P_	*A*
		Wet	Dry						
1	12.9	2.13	1.97	0.493	74	27.4	16.7	10.7	0.39
2	8.2	2.37	2.17	0.291	75.4	25.4	13.8	11.6	0.41
3	13.2	2.29	1.98	0.518	76.2	24.9	16.2	8.7	0.39
4	14.8	2.21	1.96	0.447	84.5	24.7	14.6	10.1	0.34
5	12.6	2.35	2.07	0.458	83.7	22.8	14.3	8.5	0.38

Note: *w* is water content, *ρ* is density, *e* is void ratio, *S*_r_ is saturation, *w*_L_ is liquid limit, *w*_P_ is plastic limit, *I*_P_ is plasticity index, *A* is activity index.

### Strain and culture

**Fig 3:**
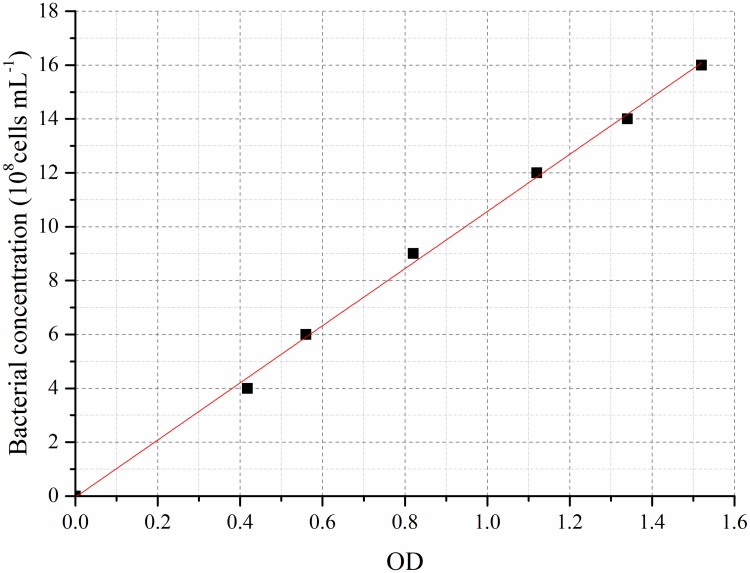
Standard curve for the relationship between the *Sporosarcina pasteurii* concentration and the OD_600_ value.

The strain of *Sporosarcina pasteurii* in the experiment was procured from the China General Microbiological Culture Collection Center (*Sporosarcina pasteurii* CGMCC 1.3687). The culture medium was a mixture of 10g/L peptone, 3g/L beef extract and 5g/L NaCl. The pH value was adjusted to 7.0. The culture medium contained in Erlenmeyer flasks was sterilized at 121°C under high pressure for 20min and taken to a sterile clean bench for tests. The activated *Sporosarcina pasteurii* was inoculated in sterile culture medium and cultivated in a vibration cultivating box with a frequency of 170r/min at a temperature of 30°C. Sampling was performed in 6 times within 24h. The plate colony counting method (S1 Fig) was adopted to count the number of bacteria and the optical density (OD_600_) value corresponding to a wavelength of 600nm was measured. The standard curve for the relationship between the *Sporosarcina pasteurii* concentration and the OD_600_ value of the bacteria solution is plotted in Fig 3.

### Healing method

In order to reduce the construction difficulty and cost in engineering practices, the homogeneity of CaCO_3_ in the MICP process shall be improved in the weak interlayer and the utilization rate of urea and Ca^2+^ shall be enhanced. In this study, after being mixed with the bacteria solution, the samples taken from weak interlayer were immersed in the cementation media. The weak interlayer was cured and healed by utilizing the MICP process between the freely penetrating cementation media and *Sporosarcina pasteurii*.

The major role of cementation media was to provide urea and Ca^2+^ for the MICP process, and to provide the nutrients for bacteria growth and reproduction. The concentration of chemical substances in the cementation media is listed in Table 3.

**Table 3:** Concentration of chemical substances in the cementation media (g/L).

NH_4_Cl	Nutrient broth	NaHCO_3_	Urea	CaCl_2_·2H_2_O
15.0	4.0	2.12	30.0	73.5

The samples were air dried and crushed, mixed with 100 mL bacteria solution, remodeling with steel mould which had a diameter of *ϕ*50mm and a height of 100mm. Polypropylene geotextile was used as the curing mold [28] (S2 Fig). The geotextile has multiple functions including anti-seepage, anti-infiltration, drainage, isolation, reinforcement, protection and sealing, which can ensure sufficient contact between the cementation media and the samples and the integrity of sample. The MICP tests were conducted in a completely stirred tank reactor (S3 Fig). The reactor mainly included an organic glass box to contain samples and cementation media, a magnetic stirrer at the bottom to keep the solution uniform, and an air pump to provide oxygen for bacteria.

The bacterial concentration directly affects the hydrolysis speed of urea, which will further affects the precipitation process of CaCO_3_. In order to study the effects of bacterial concentrations on the recovery of weak interlayer, 6 repairing concentrations of bacteria were prepared with a concentration of Ca^2+^ in the binding liquid of 0.5mol/l. It lasted 30 d in the binder and 7 d drying at room temperature before further tests.

### Mechanical strength test

**Fig 4:**
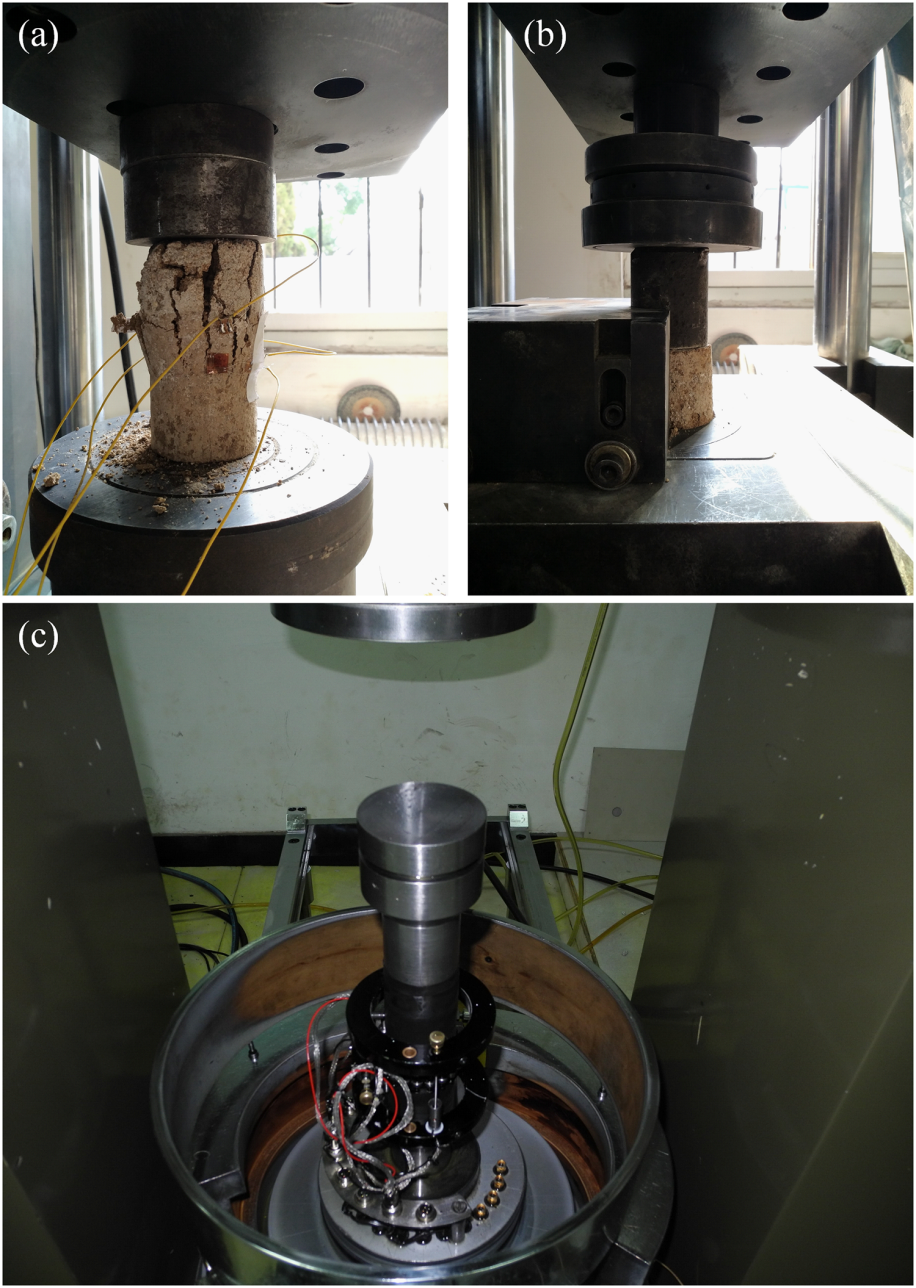
Laboratory tests showing (a) uniaxial compression tests, (b) direct shear tests, and (c) triaxial compression tests.

In order to study the repairing effect of MICP on the mechanical properties of weak interlayer. In this study, the uniaxial compression test, direct shear test and triaxial compression test (Fig 4) were performed on the specimens of different concentrations of bacteria. The mechanical parameters of the soft interlayer was obtained. The variation of the mechanical parameters with the concentration of the bacteria liquid was analyzed.

### CaCO_3_ content Test

The content of CaCO_3_ in the weak interlayer is directly related to the repairing effect and mechanical properties of the test piece, so it is necessary to test the content of CaCO_3_. The repaired weak interlayer test piece was ground into a powder by a mortar and placed in an oven to be dried, and the weight at this time was recorded as *W*_1_. And then put it into a 0.1mol/ml HCl solution to soak and wash, until no more bubbles. Then after washing with deionized water, filtration, drying, the weight measured as *W*_2_. The difference between the *W*_1_ and *W*_2_ (*W*_1_-*W*_2_) is the weight of CaCO_3_. The difference value divided by the original total weight *W*_1_, recorded as WT, was presenting the CaCO_3_ percent content. As shown in the formula (1).

$$\text{WT}=\frac{W_{1}-W_{2}}{W_{1}}\text{\%}$$ (1)

## Results and discussion

### SEM and EDS analysis

**Fig 5:**
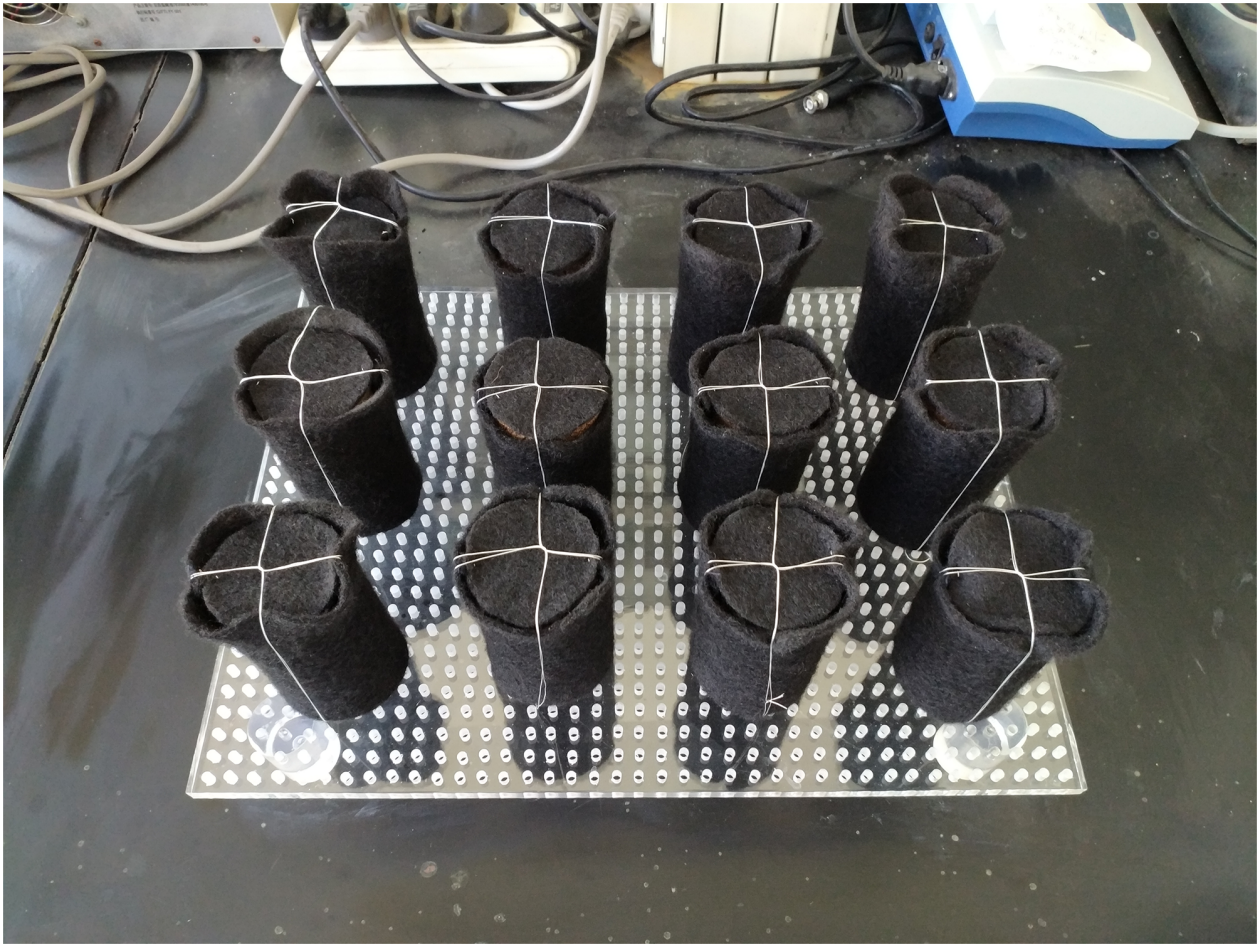
White precipitates on geotextile mold around samples cured by MICP.

**Fig 6:**
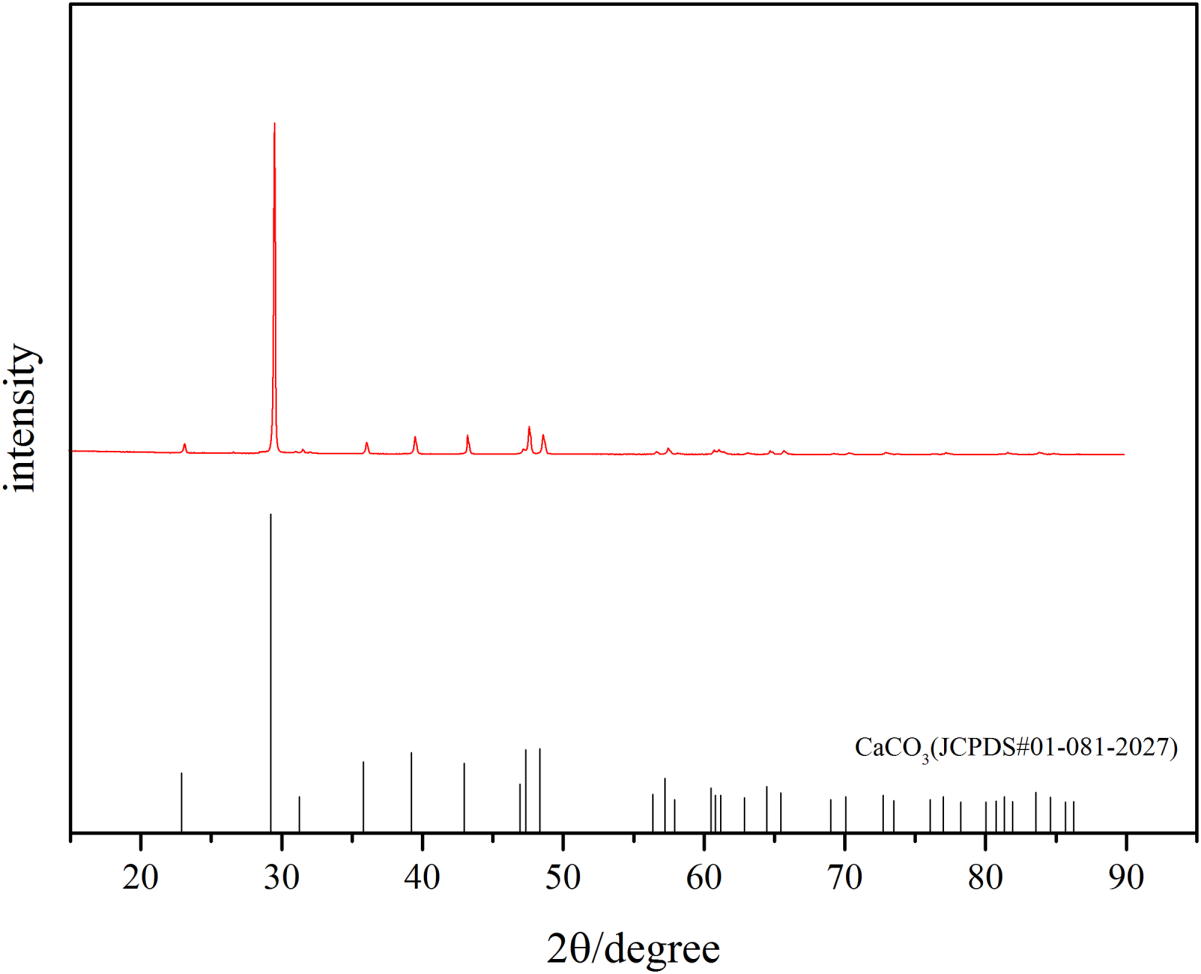
The XRD spectrum of white precipitates on geotextile.

The concentration of the bacterial solution was about 9.5×10^8^ /mL when the OD_600_ value was 0.9, according to the concentration of the bacterial solution and the OD_600_ standard curve. The SEM and EDS analysis were performed with the sample. Take out the specimen after repairing for 30 days, continuous white precipitates were clearly visible on the geotextile mold, as shown in Fig 5. The XRD analysis was conducted on the precipitates as shown in Fig 6. It is noticed that the spectra obtained from precipitates agrees well with the standard XRD spectra of CaCO_3_ (JCPDS#01-081-2027), showing that the precipitates are CaCO_3_ and its form is calcite.

**Fig 7:**
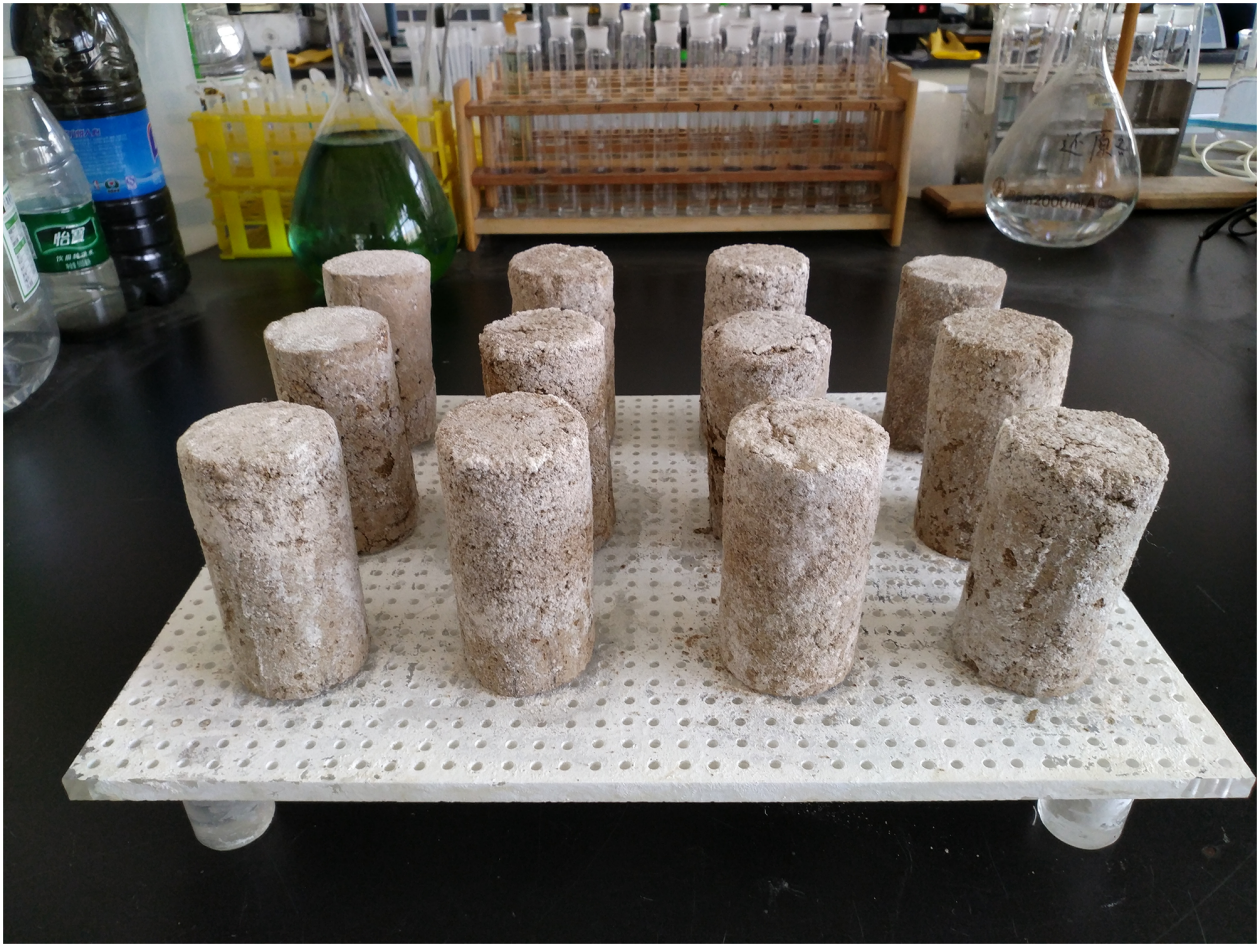
Cured samples after mold removal.

**Fig 8:**
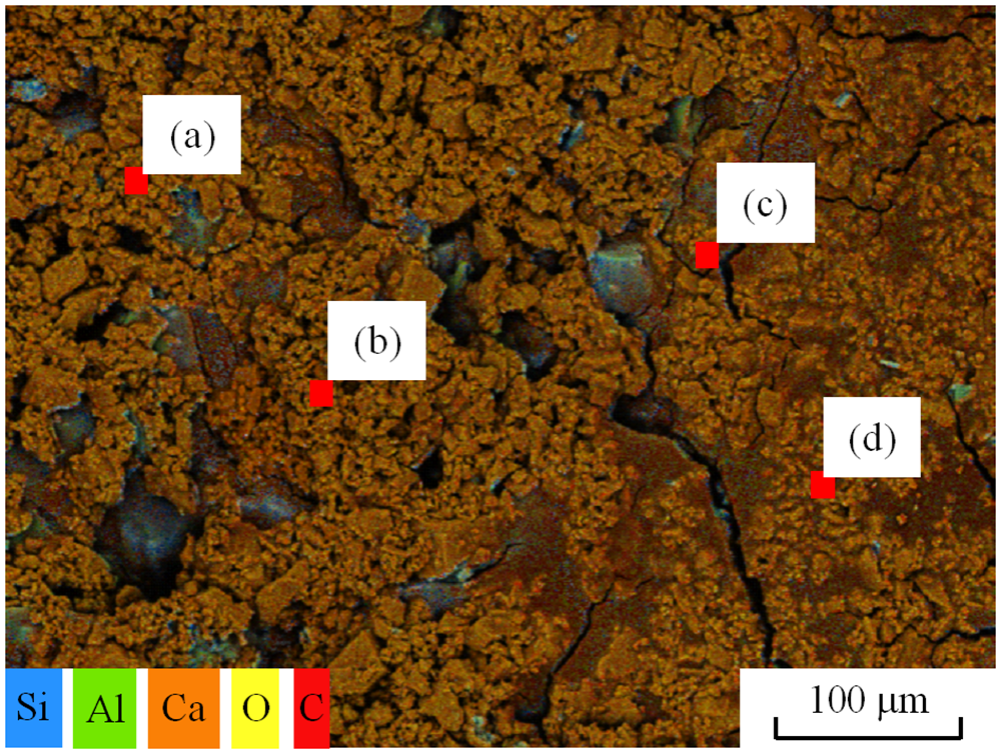
The EDS layered image of MICP-treated sample.

**Fig 9:**
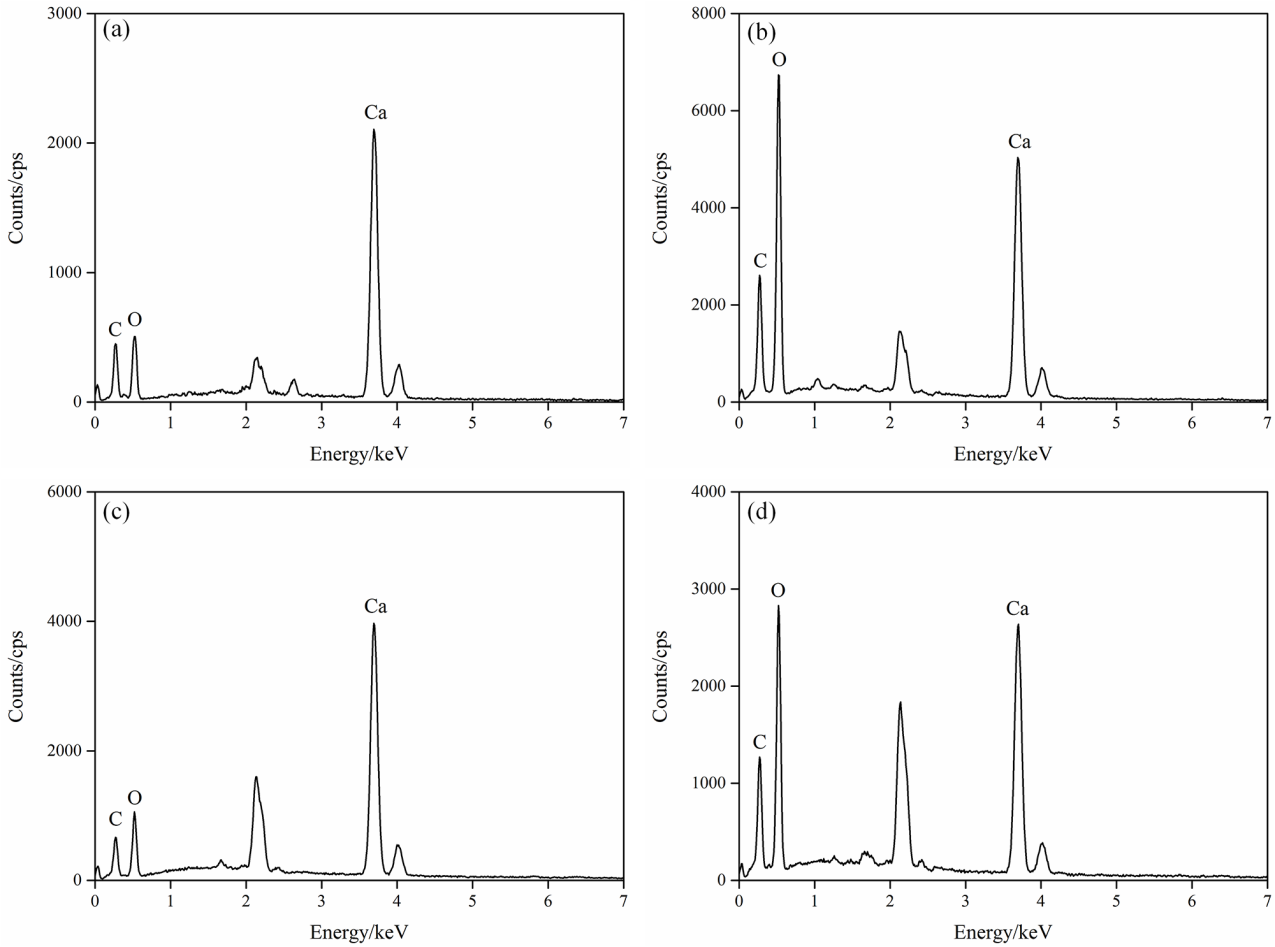
EDS analysis of the precipitates on the sample surface from (a) region a, (b) region b, (c) region c, and (d) region d shown in Fig 8.

The cured samples were then air-dried at room temperature for 7d before the geotextile mold was removed. The samples were cylindrical and very hard, as shown in Fig 7. In order to further analyze the precipitation structure and composition of CaCO_3_ produced in the MICP process, the EDS analysis under scanning electron microscopy (SEM) was performed for the precipitates and the morphology of the particles was observed. As shown in Fig 8, the EDS layered image presents the distribution of Si, Ca, C and O elements in the test area. The contact surfaces between the weak interlayer particles and calcite are clearly visible. The EDS energy spectrum analysis was performed for the areas a-d, as illustrated in Fig 9. The energy spectra indicates that the precipitates mainly consist of three elements, namely, C, O and Ca. Hence, it is further verified that CaCO_3_ crystals were produced during the MICP process.

**Fig 10:**
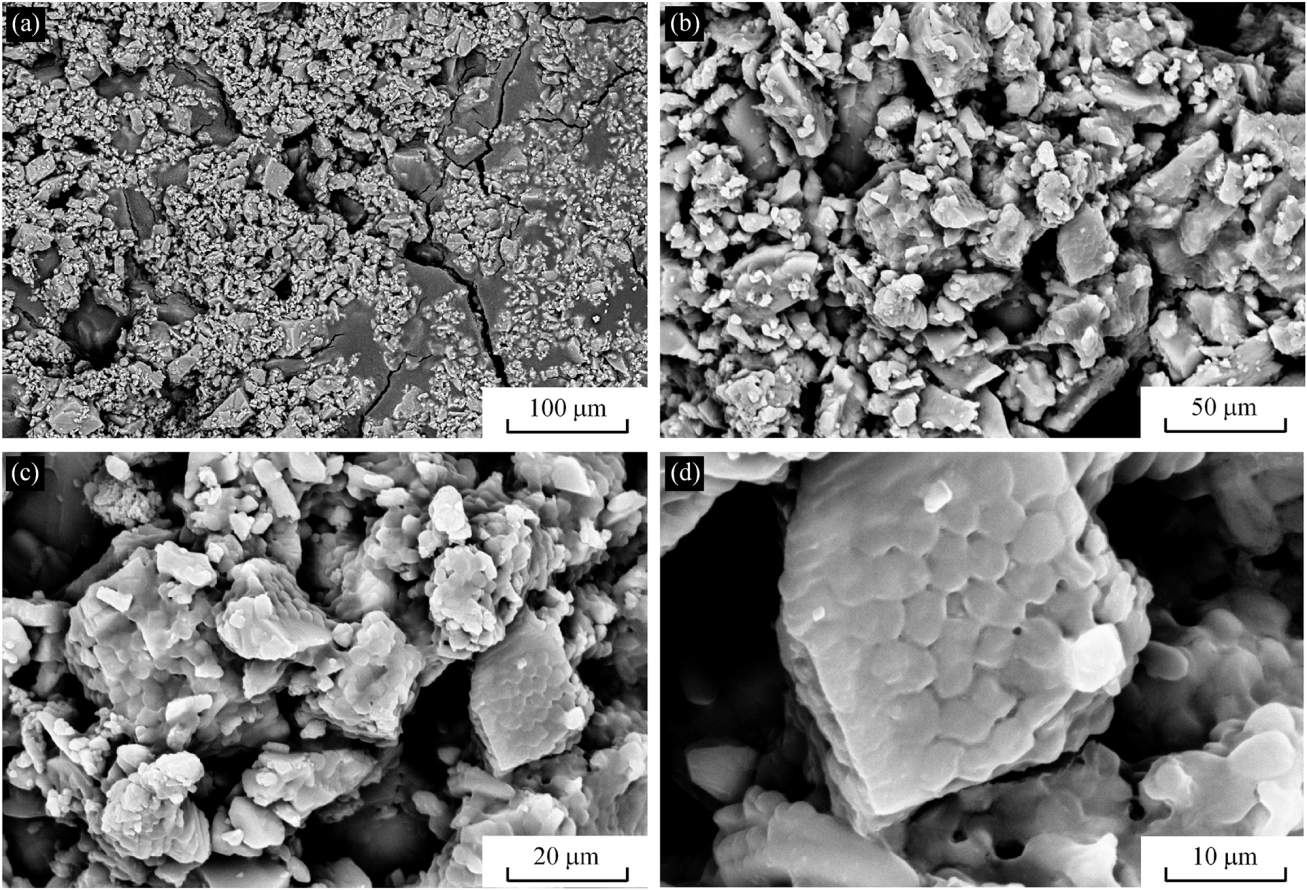
Scanning electron micrographs of MICP-treated samples. (a) and (b) are CaCO_3_ particles and the interlayer particles. (c) and (d) are CaCO_3_ particles.

The morphology of CaCO_3_ particles was observed by SEM. As can be seen from Fig 10a and 10b, the CaCO_3_ particles and the interlayer particles are cemented together in the cured sample. Due to high concentration of water-soluble organic matters in the reactor, the growth of CaCO_3_ crystals is obviously controlled by the organic protein macromolecules and multistage growth and aggregation occur. Its crystals were irregular fish scale-shaped cubes. As shown in Fig 10c and 10d, the crystal edges and corners are clear and the crystals grow well, with size ranging between 5~20*μ*m.

### Mechanical test results and analysis

**Fig 11:**
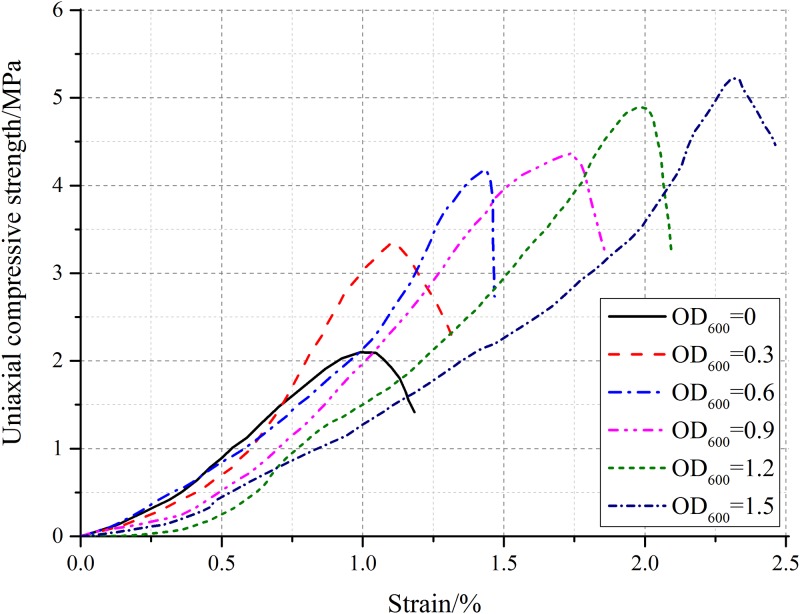
Variation of uniaxial compressive strength with strain obtained by uniaxial compressive tests on typical samples.

**Fig 12:**
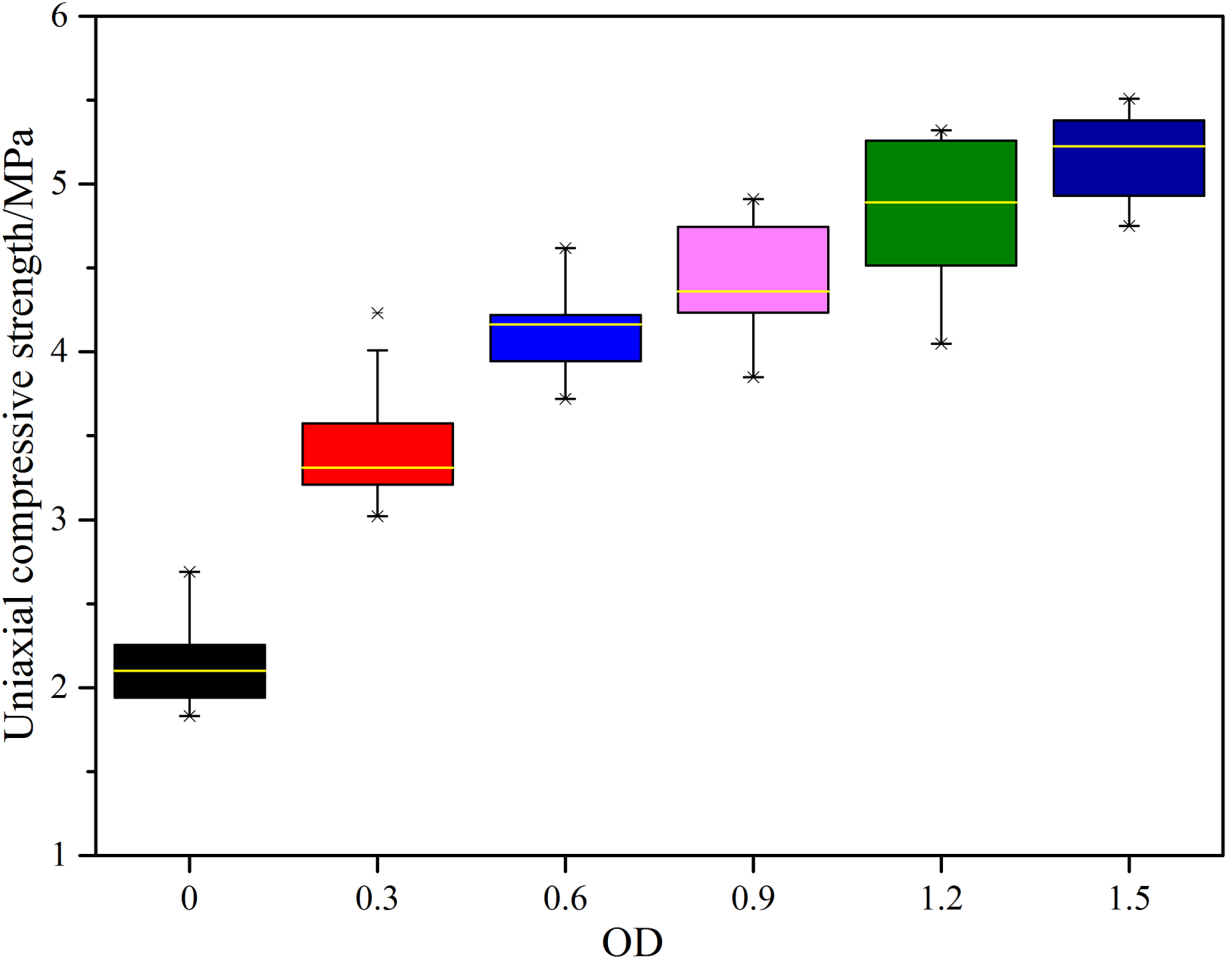
Box plot for uniaxial compressive strength.

Fig 11 shows the variation of uniaxial compressive strength with strain obtained by uniaxial compression tests on typical samples under six different bacterial concentration. As MICP is a complex biochemical process, the experiment process is susceptible to external factors. In order to intuitively present the data distribution, the uniaxial compressive strength of all the samples cured under six different bacteria concentration is illustrated by box plot, as shown in Fig 12. As can be seen from Figs 11 and 12, although the uniaxial compressive strength of samples cured under a certain bacteria concentration changes slightly in a range, it generally increases with increasing bacteria concentration. When the OD_600_ value increasing from 0.3 to 0.6, the uniaxial compressive strength is obviously increased by 60% and 90%, respectively. When the OD_600_ value further increasing to 0.9, 1.2 and 1.5, the uniaxial compressive strength is increased by 108%, 133% and 149%, respectively. The increasing trend tends to be gentle. It is shown that the engineering performance of cured samples were effectively enhanced by bacteria concentration increasing.

**Fig 13:**
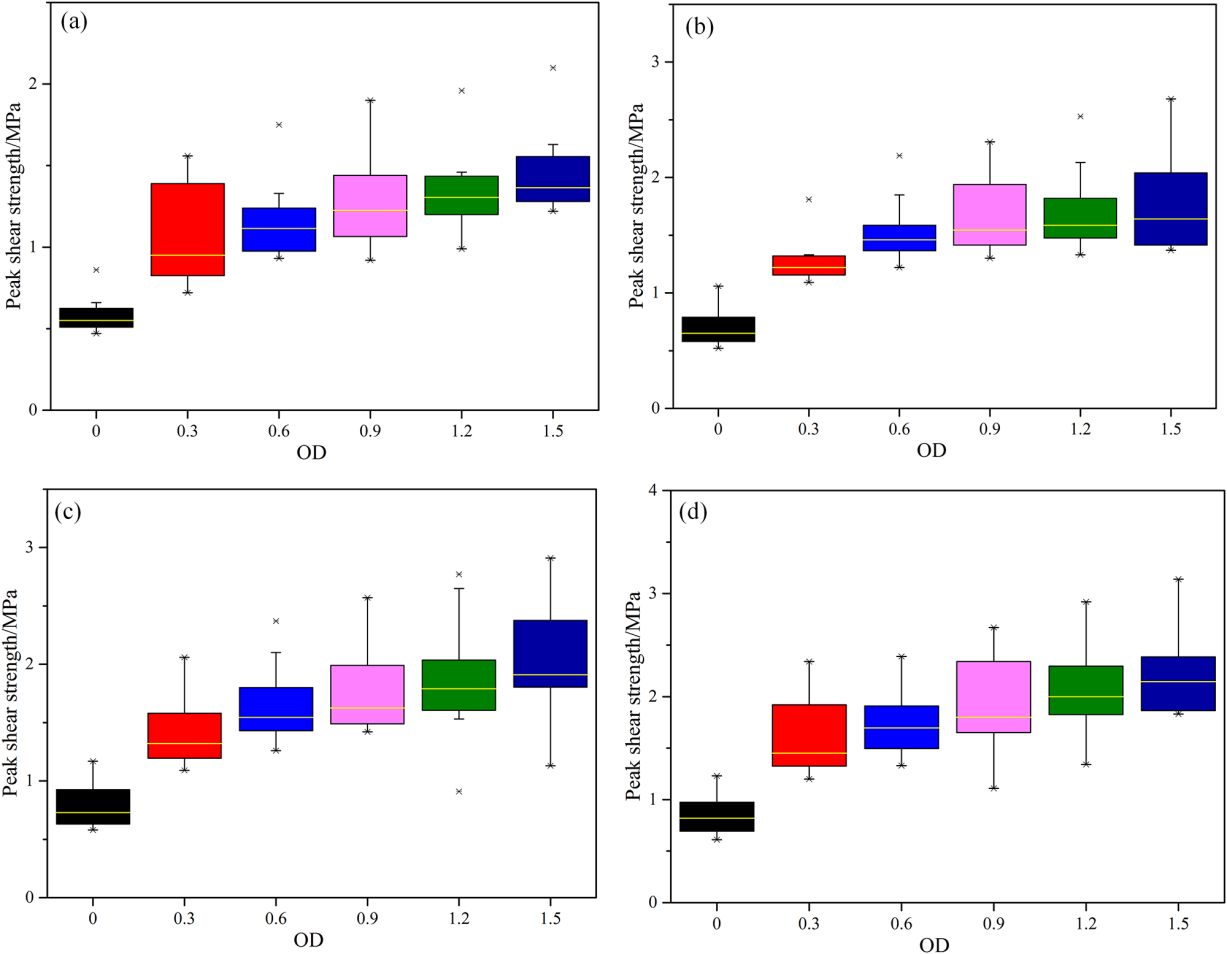
Box plot of peak shear strength of cured samples under different normal stresses. (a) *σ*_n_ = 0.5 MPa, (b) *σ*_n_ = 1 MPa, (c) *σ*_n_ = 1.5 MPa, and (d) *σ*_n_ = 2 MPa.

In order to investigate the effect of MICP on improving the shear property of weak interlayer, shear tests were performed on cured samples under 4 different normal stresses (0.5 MPa, 1 MPa, 1.5 MPa and 2 MPa). Firstly, the bearing plate of normal stress was manually adjusted to a position near the specimen. Then, the target normal stress of testing machine was achieved with a loading speed of 20 kN per minute. Next, manually adjusted the bearing plate of shear stress to a position close to specimen. Finally, tested on specimen with a loading speed of 20 kN per minute until it totally damaged. The test results are shown in Fig 13. By comparing the four box plots, it can be found that the peak shear strength of cured samples is improved obviously and increases continuously with increasing bacteria concentration. Taking the sample under a normal stress of 1.5 MPa as an example (Fig 13c), when the OD_600_ value increases from 0.3 to 0.6, the peak shear strength of cured samples is increased by 81% and 112%, respectively. The increasing trend is obvious. When the OD_600_ value further increases to 0.9, 1.2 and 1.5, the peak shear strength of cured samples is increased by 123%, 145% and 162%, respectively. The increasing trend becomes gentle. By comparison, the effect of bacterial concentration on peak shear strength is less than the effect on uniaxial compressive strength. This is because the cementation effect of CaCO_3_ on the weak interlayer particles is limited. The effect of CaCO_3_ is mainly embodied in the pressure bearing effect, and the cohesion and friction angle of the test piece are less repaired.

**Fig 14:**
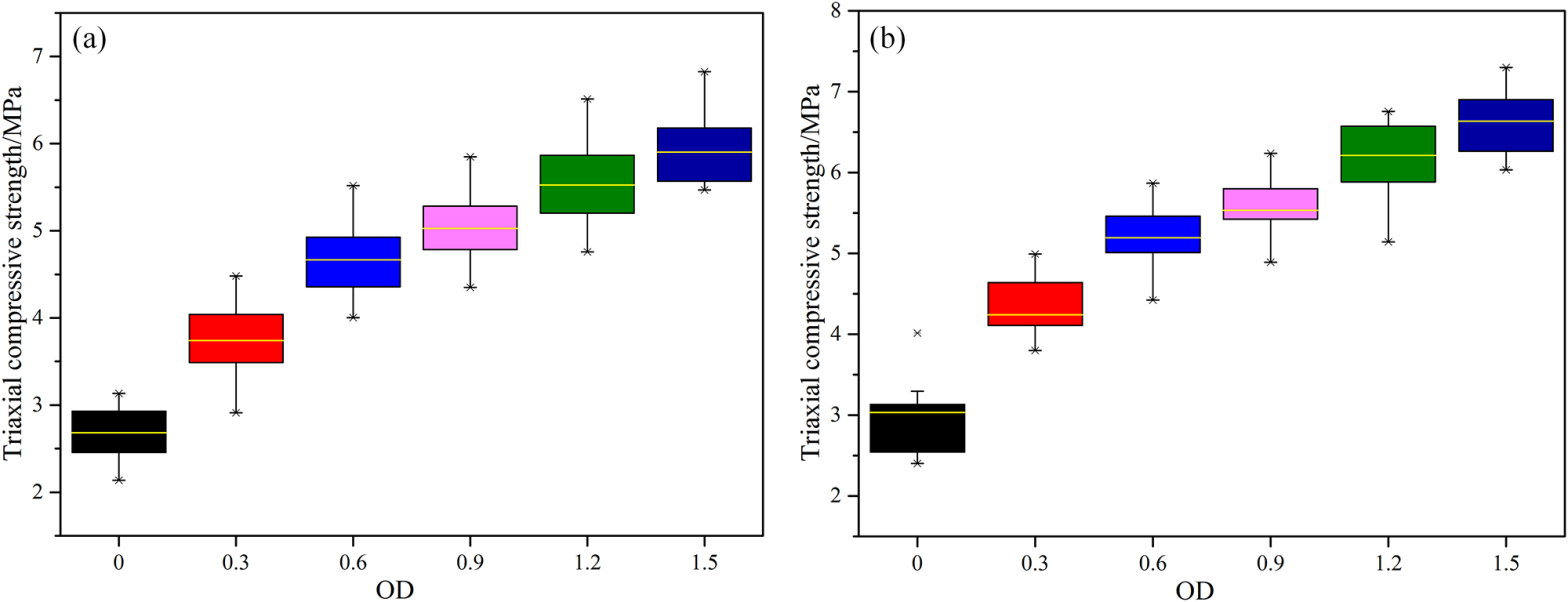
Box plot of triaxial compressive strength of cured samples under different confining pressures. (a) *σ*_3_ = 1 MPa and (b) *σ*_3_ = 2 MPa.

In order to further analyze the effect of MICP on improving the mechanical performance of weak interlayer in engineering practices, conventional triaxial compression tests were conducted on cured samples under two different confining pressures (1 MPa and 2 MPa). The results are presented by box plot in Fig 14. The results indicate that the triaxial compressive strength of cured samples is improved obviously and increases with increasing confining pressure. Taking the sample under a confining pressure of 2 MPa as an example (Fig 14b), when the OD_600_ value increases from 0.3 to 0.6, the triaxial compressive strength of cured samples is increased by 40% and 71%, respectively. The increasing trend is obvious. When the OD_600_ value further increases to 0.9, 1.2 and 1.5, the triaxial compressive strength of cured samples is increased by 83%, 105% and 119%, respectively. The increasing trend tends to be gentle.

As a summary, for healing of weak interlayer by *Sporosarcina pasteurii* induced calcium carbonate precipitation, when the bacteria concentration OD_600_ is smaller than 0.6, the mechanical parameters of healed weak interlayer are improved remarkably; when the OD_600_ value is greater than 0.6, the increment rate of mechanical parameters gradually decreases and tends to be stable. Therefore, the test results corresponding to a bacteria concentration OD_600_ of 1.5 shall be taken as the extremums of various mechanical parameters for the healed weak interlayer.

### Analysis of CaCO_3_ content test results

**Fig 15:**
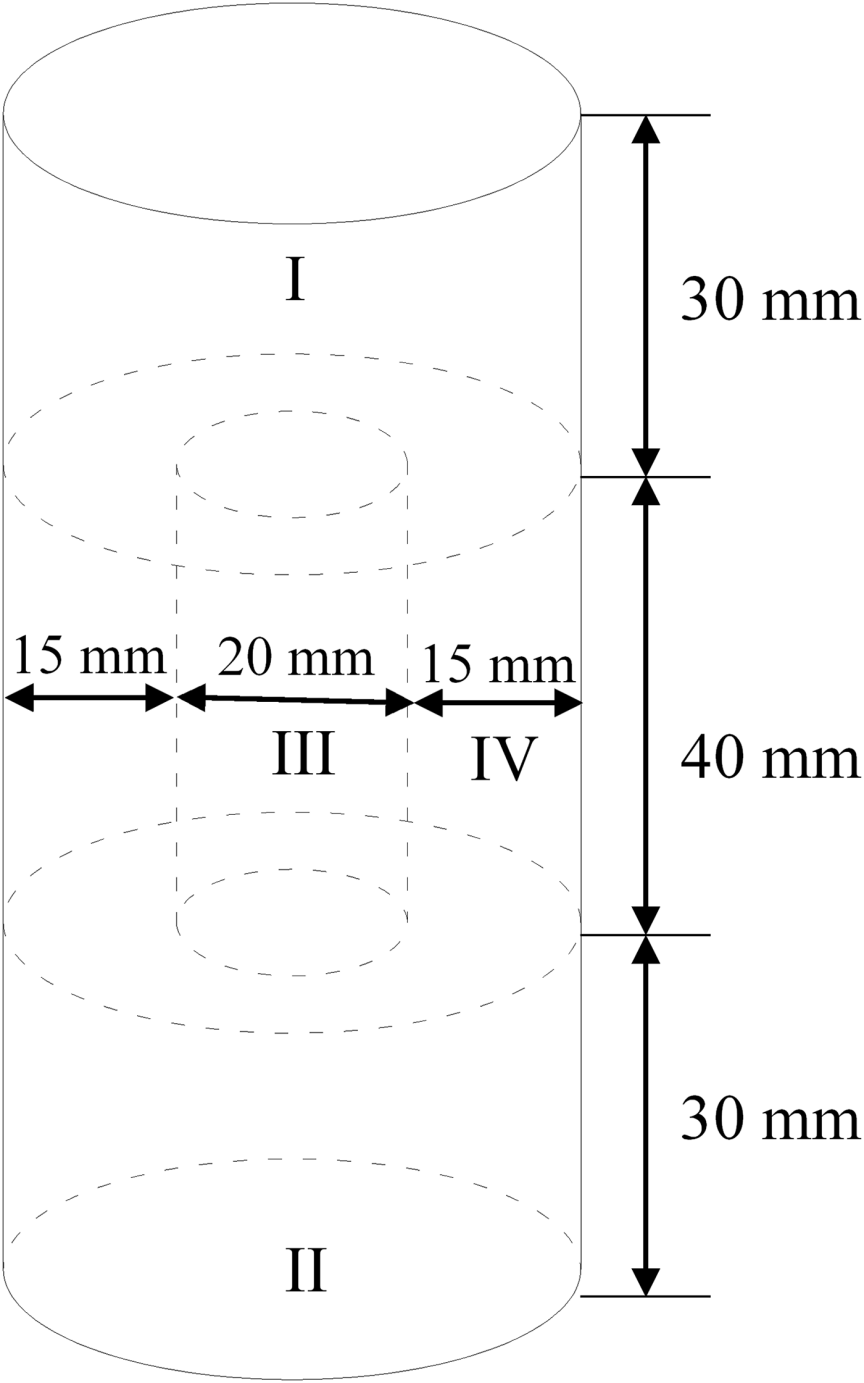
Size of the four areas in the analysis of CaCO_3_ content in the specimen.

**Fig 16:**
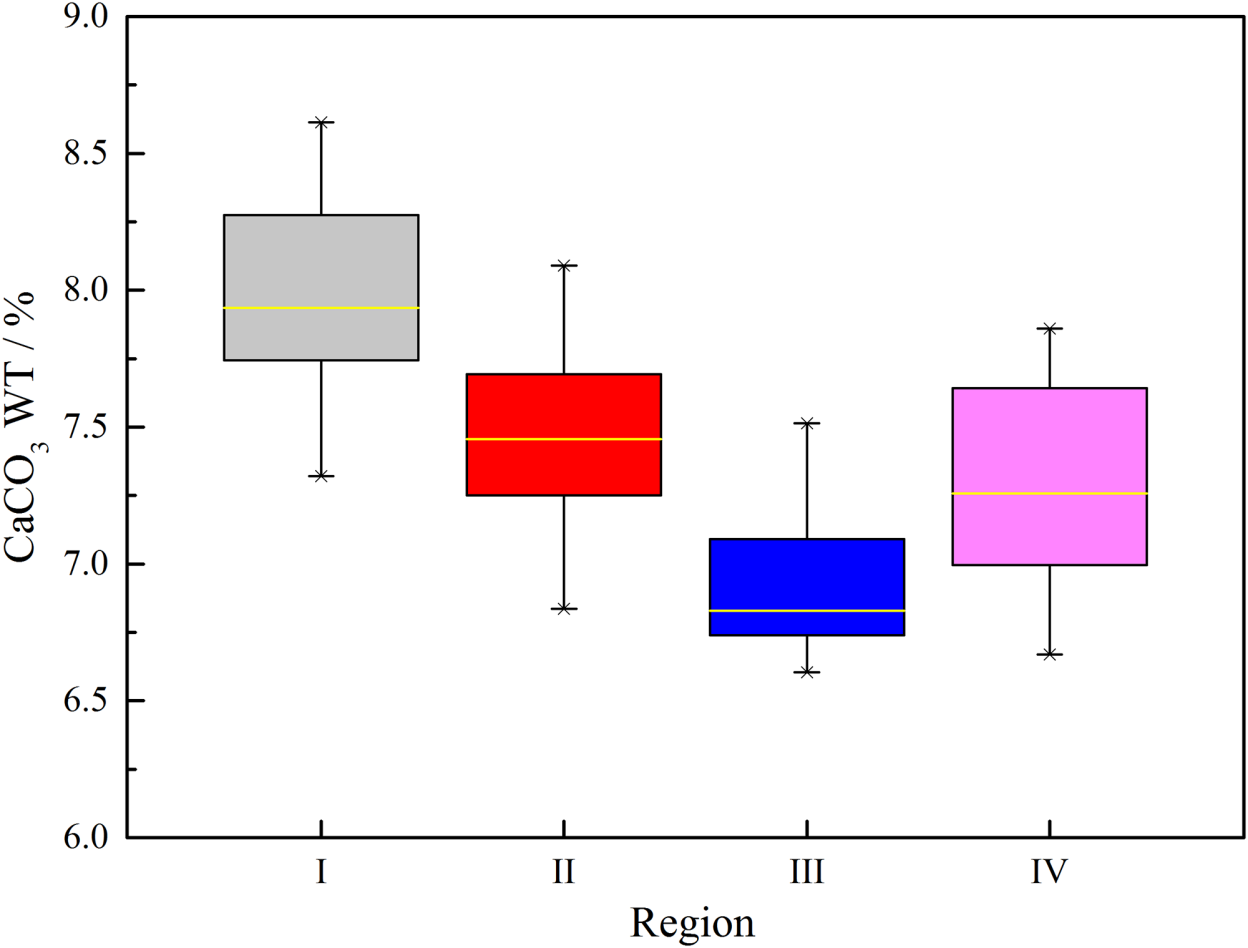
Box plot for CaCO_3_ content of the specimen after healing at OD_600_ 0.9.

The content of CaCO_3_ was tested by using the repaired sample with OD_600_ as 0.9. In order to fully consider the CaCO_3_ content in different positions in the test piece, the test piece is divided into four areas I~IV, as shown in Fig 15. The CaCO_3_ content of the four regions was tested separately and plotted in a box drawn, as shown in Fig 16. It can be seen from the figure that the change of CaCO_3_ content in each region is 6.60%~8.61%, and the numerical values are not much different, among which the content of I, II and IV is higher, and the content of CaCO_3_ in III is the lowest. This is because the weak interlayer particles in zone I, zone II and zone IV are located at the top, bottom and outside, and can be fully contacted by geotextile and repair fluid. The zone III is located in the center of the specimen. After the formation of CaCO_3_ in other areas, some of the particle clearance will be blocked, so that the contact between the weak interlayer and the repair fluid in zone III is weakened. Although there are slight differences in the content of CaCO_3_ in four regions, in general, it can be considered that the uniformity of the specimen has reached a relatively good state. Therefore, in the subsequent experiments, the specimen can be used as a whole, and the CaCO_3_ content can be calculated by using the formula (1).

**Fig 17:**
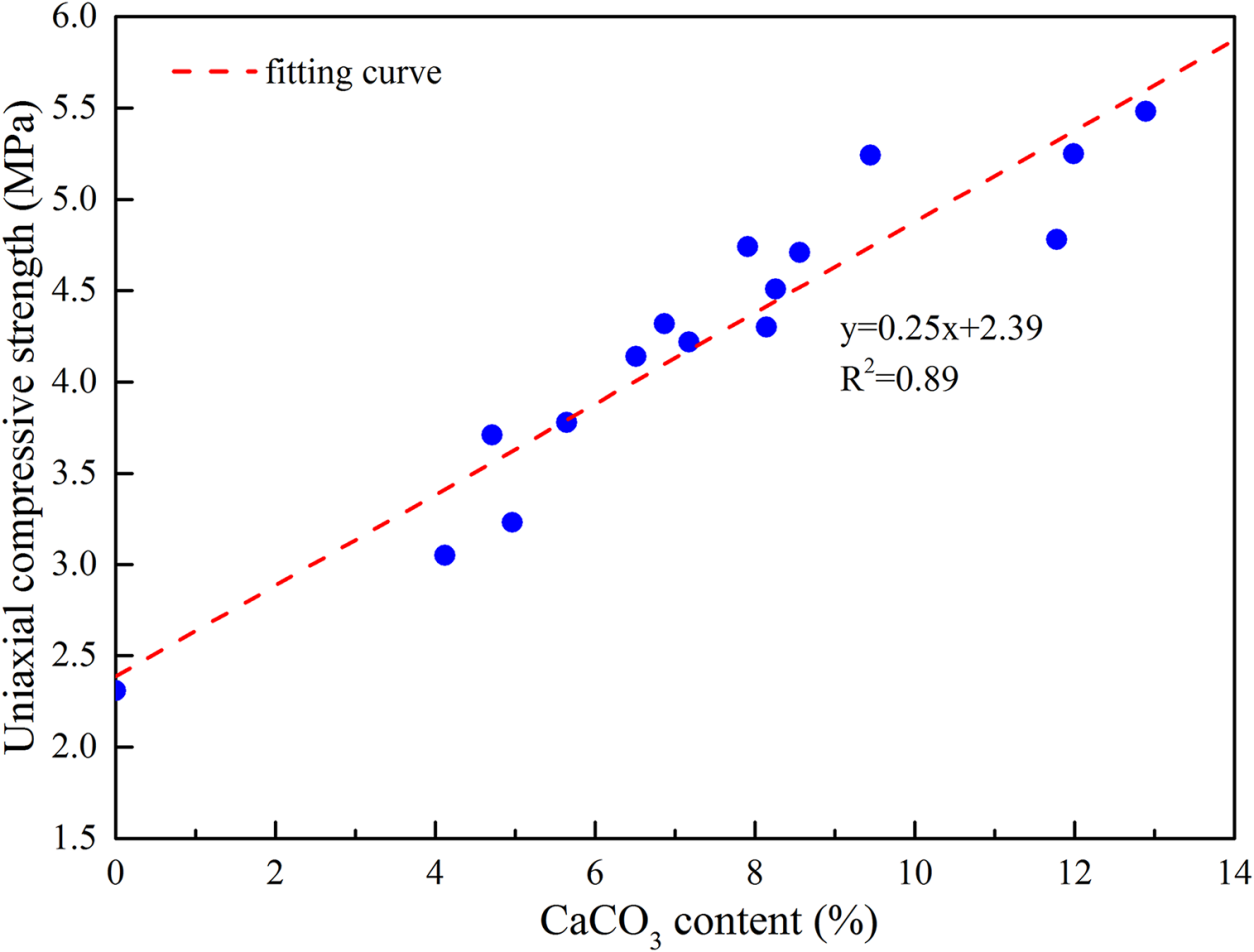
Relationship between uniaxial compressive strength and CaCO_3_ content.

**Fig 18:**
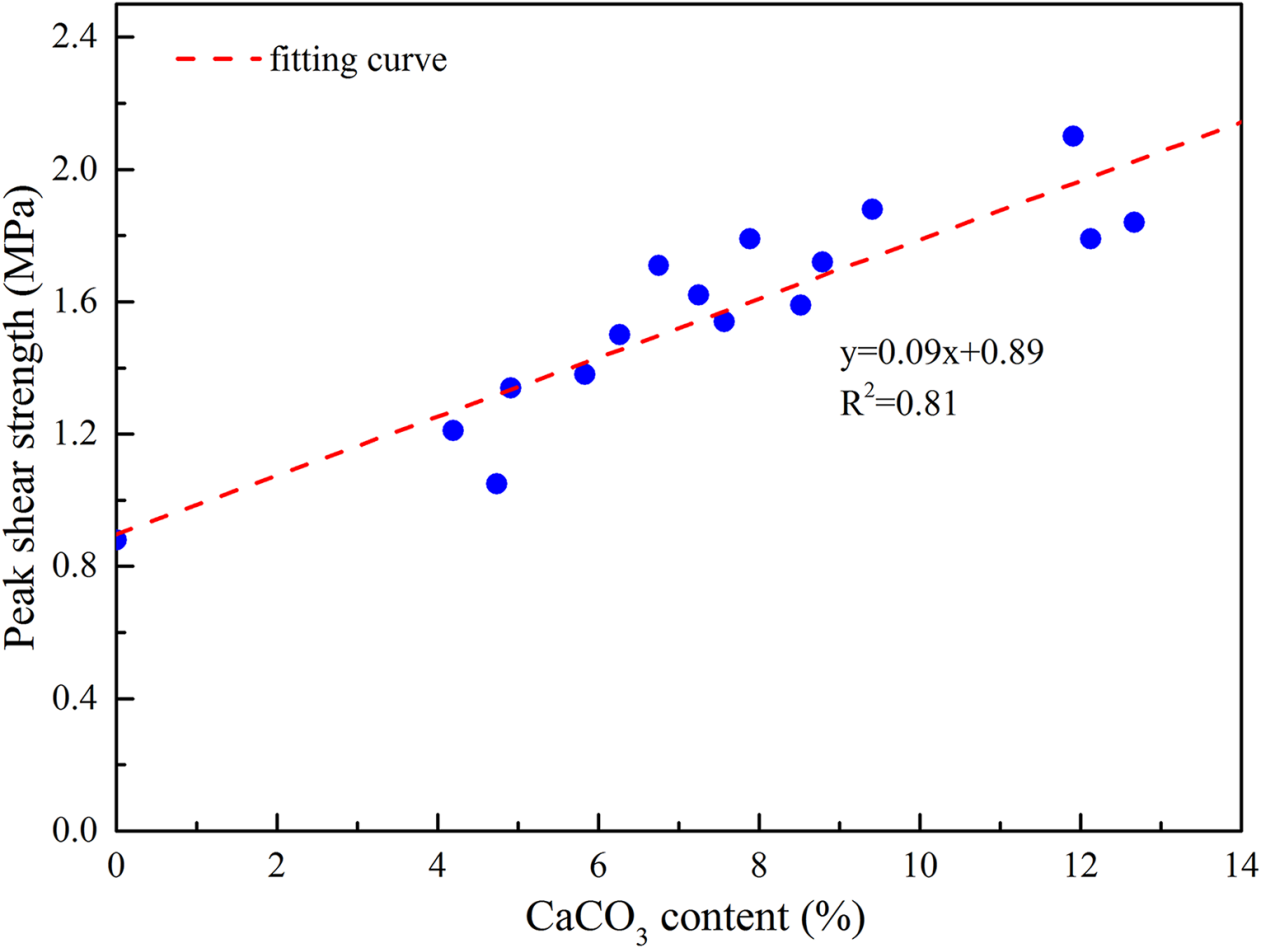
Relationship between peak shear strength and CaCO_3_ content under normal stress 1.5MPa.

**Fig 19:**
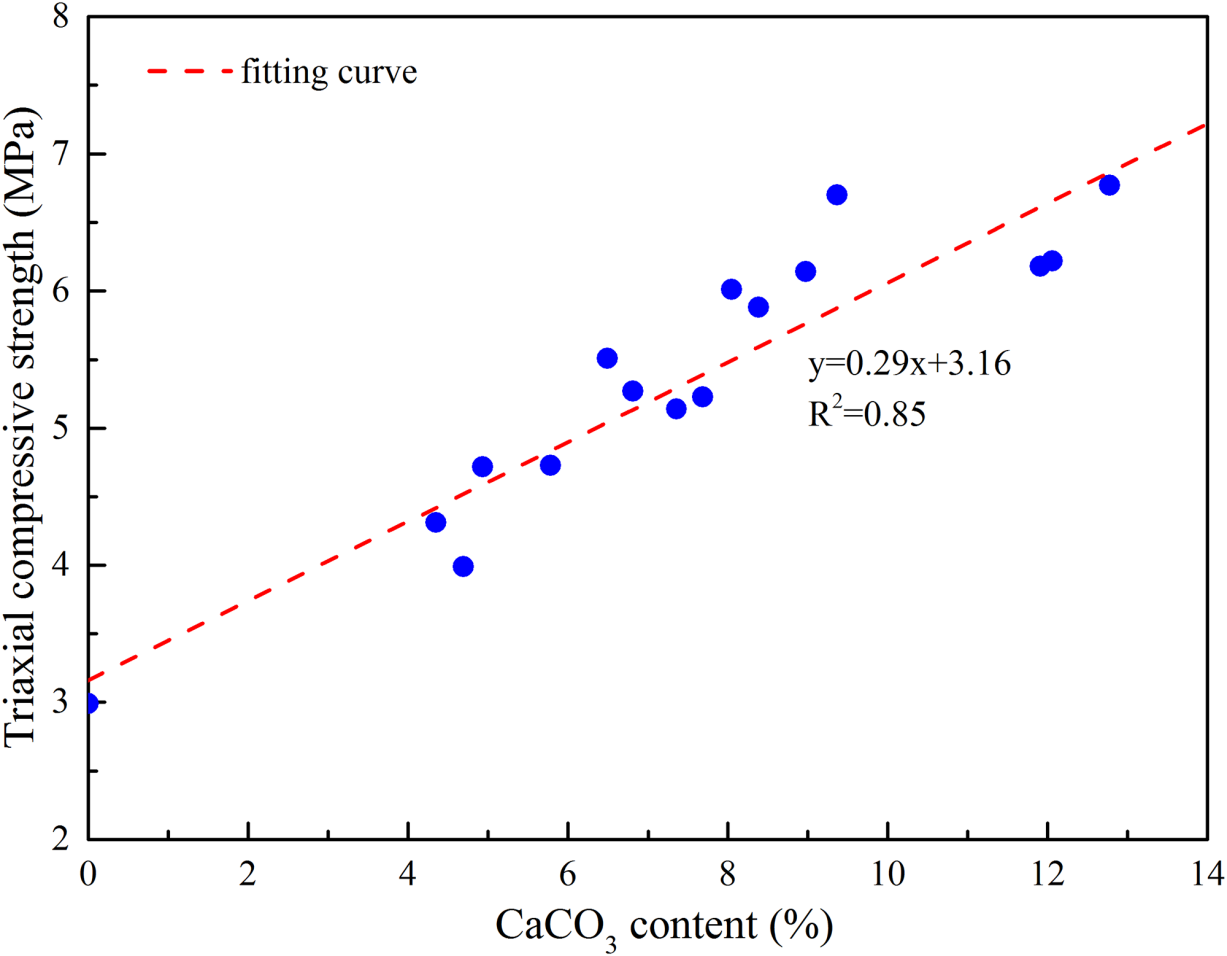
Relationship between triaxial compressive strength and CaCO_3_ content under confining pressure 2MPa.

After the laboratory mechanics experiment was finished, three specimens were selected as the CaCO_3_ content test samples at each bacterial concentration, and the test results were shown in Table 4. As can be seen from the analysis in Table 4, it is shown that with the increase of bacterial concentration, the ability of bacteria to decompose urea is increasing, so the content of the specimen CaCO_3_ is increasing. The uniaxial compressive strength, peak shear intensity(*σ*_n_ = 1.5MPa), triaxial compressive strength(*σ*_3_ = 2MPa) and CaCO_3_ content are plotted separately, as shown in Figs 17, 18 and 19. As we can see from the figure that the mechanical strength of the test piece increases linearly with the increase of CaCO_3_ content. This is because the CaCO_3_ induced by bacteria can not only bond the weak interlayer particles together, but also increase the integrity of the test piece. At the same time, the presence of CaCO_3_ also plays a role in improving the mechanical strength. In conclusion, increasing the concentration of bacteria can increase the CaCO_3_ content of the weak interlayer and improve the mechanical strength of the weak interlayer.

**Table 4:** Concentration of chemical substances in the bonding solution (g/L).

OD_600_	0	0.3	0.6	0.9	1.2	1.5
CaCO_3_ WT %	0	4.12–4.96	5.64–6.87	7.18–8.15	8.26–9.45	11.78–12.89

## Conclusions

In this study, *Sporosarcina pasteurii* was employed for microbial induced calcium carbonate precipitation (MICP) to heal the weak interlayer at Baihetan Hydropower Station on the lower reach of Jinsha River, China. With observations by naked eyes, XRD and SEM, it is shown that the MICP based healing technique for weak interlayer is feasible. The variations of uniaxial compressive strength, peak shear strength and triaxial compressive strength of cured samples with bacteria concentration were obtained by mechanical tests. The results in this study indicate that the MICP based healing technique for weak interlayer can not only enhance the integrity of rock masses, but also improve the mechanical properties of weak interlayer. The maximum uniaxial compressive strength, peak shear strength and triaxial compressive strength can be increased by 149%, 162% and 119%, respectively. This healing technique can provide a new research method and idea for rock mass reinforcement.

## Supporting information

S1 Fig Counting by plate colony counting method. (TIF)

S2 Fig Samples in geotextile molds. (TIF)

S3 Fig The reactor. (TIF)
