# Supplementary figures and images for: Study on healing technique for weak interlayer and related mechanical properties based on microbially-induced calcium carbonate precipitation
Source: PLoS One. 2018 Sep 13;13(9):e0203834. doi: 10.1371/journal.pone.0203834 (PMC6136771; doi:10.1371/journal.pone.0203834)

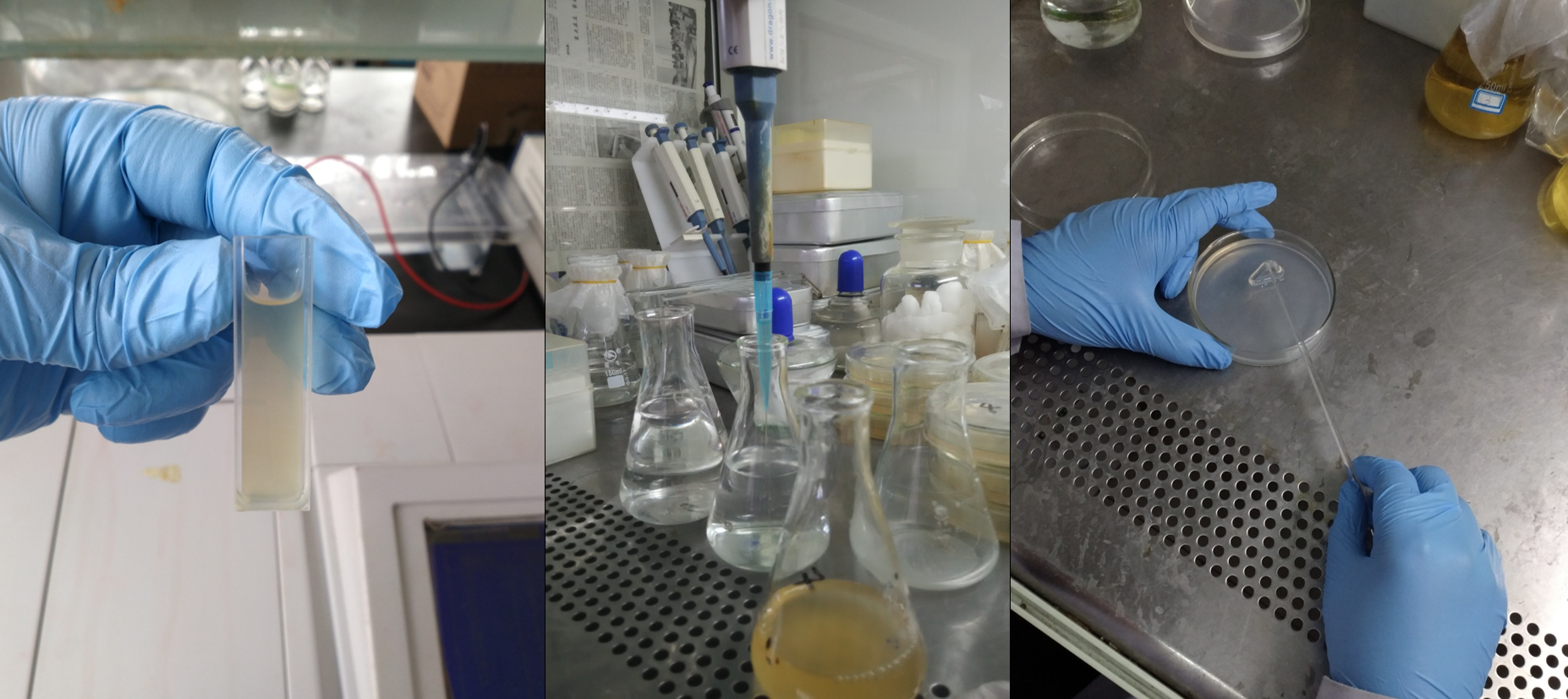

Supplement: S1 Fig — (TIF) [file pone.0203834.s001.tif]

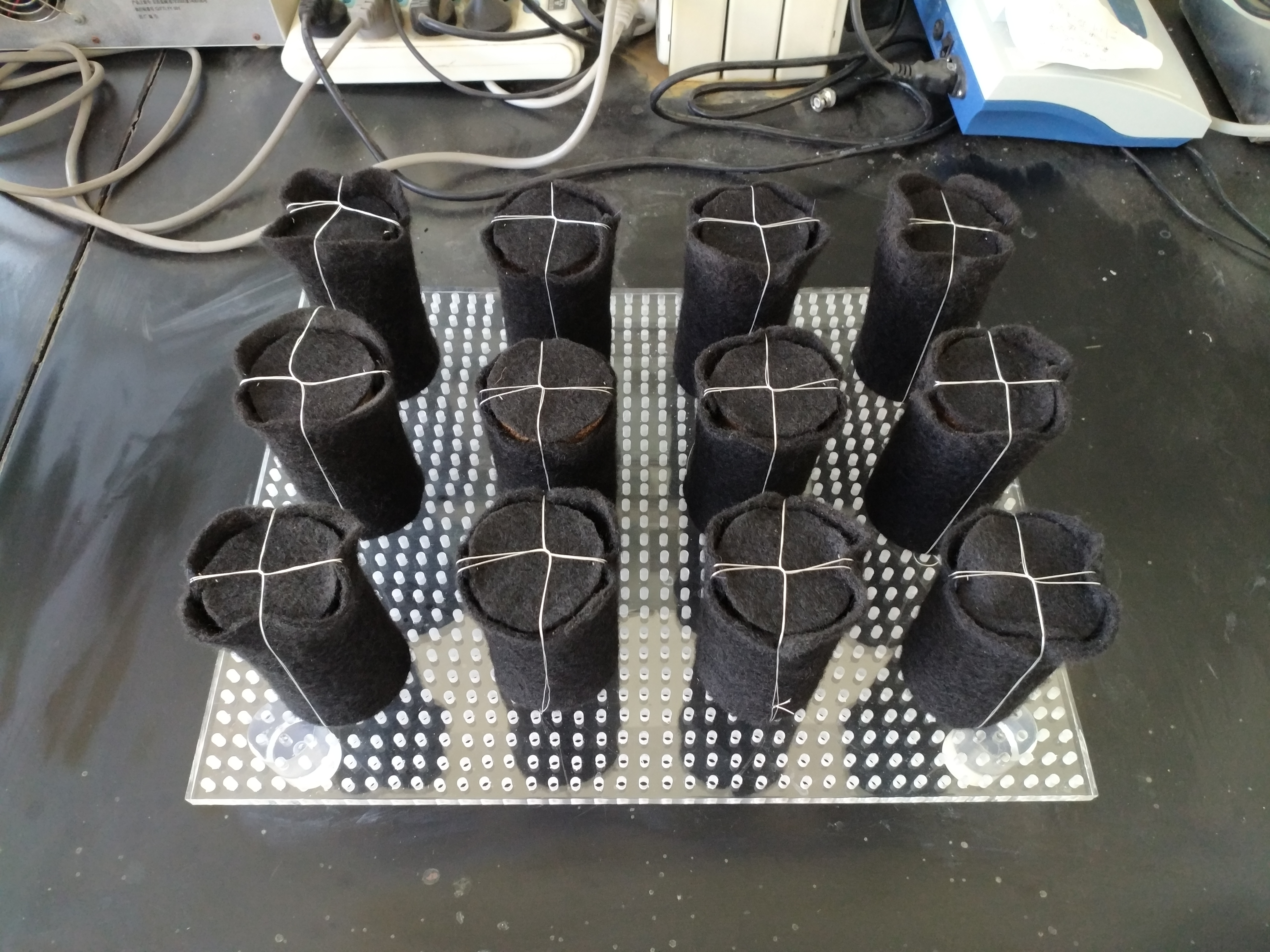

Supplement: S2 Fig — (TIF) [file pone.0203834.s002.tif]

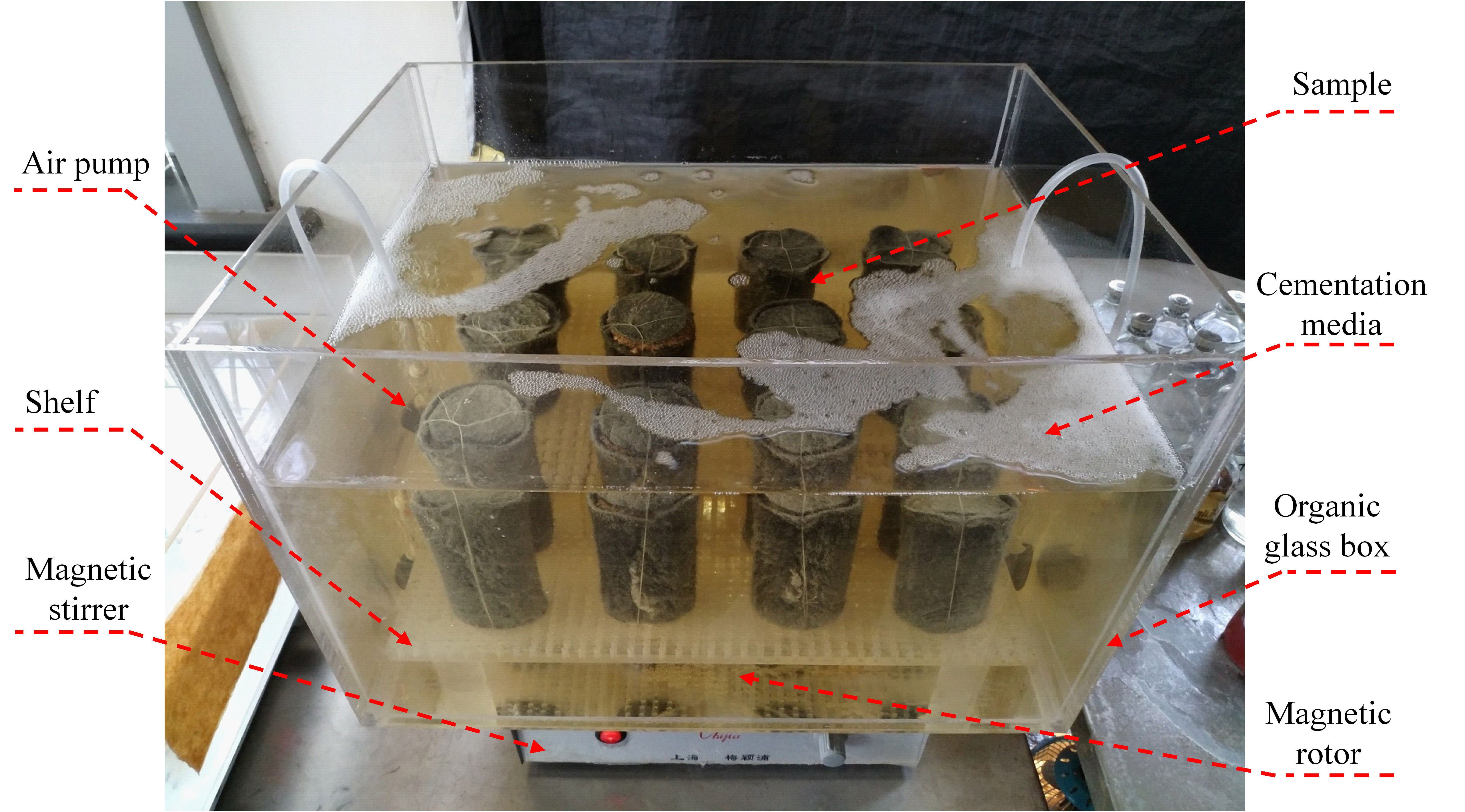

Supplement: S3 Fig — (TIF) [file pone.0203834.s003.tif]
